# Burden of ischemic stroke attributable to a high red meat diet in China, 1990–2019: analysis based on the 2019 Global Burden of Disease Study

**DOI:** 10.3389/fnut.2024.1384023

**Published:** 2024-06-06

**Authors:** Shuai Jin, Kaide Xia, Baofei Sun, Lang Xie, Haiwang Zhang

**Affiliations:** ^1^School of Biology and Engineering, School of Health Medicine Modern Industry, Guizhou Medical University, Guiyang, China; ^2^Guiyang Maternal and Child Health Care Hospital, Guiyang Children's Hospital, Guiyang, China; ^3^Key Laboratory of Human Brain Bank for Functions and Diseases of Department of Education of Guizhou Province, Guizhou Medical University, Guiyang, China; ^4^Department of Preventive Health Care, Bijie Hospital of Zhejiang Provincial People's Hospital, Bijie, China; ^5^Department of Neurosurgery, Guizhou Provincial People's Hospital, Guiyang, China

**Keywords:** diet high in red meat, global burden of disease, China, ischemic stroke, annual percentage changes

## Abstract

**Background:**

The burden of ischemic stroke (IS) linked to high consumption of red meat is on the rise. This study aimed to analyze the mortality and disability-adjusted life years (DALYs) trends for IS attributed to high red meat intake in China between 1990 and 2019 and to compare these trends with global trends.

**Methods:**

This study extracted data on IS attributed to diets high in red meat in China from 1990 to 2019 from the Global Burden of Disease Study (GBD) database. Key measures, including mortality, DALYs, age-standardized mortality rates (ASMR), and age-standardized DALYs rates (ASDR), were used to estimate the disease burden. The estimated annual percentage change and joinpoint regression models were employed to assess the trends over time. An age-period-cohort analysis was used to assess the contribution of a diet high in red meat to the age, period, and cohort effects of IS ASMR and ASDR.

**Results:**

Between 1990 and 2019, deaths and DALYs from IS attributed to a diet high in red meat in China, along with corresponding age-standardized rates, significantly increased. The overall estimated annual percentage change for the total population and across sex categories ranged from 1.01 to 2.08. The average annual percentage changes for overall ASDR and ASMR were 1.4 and 1.33, respectively, with male ASDR and ASMR average annual percentage changes at 1.69 and 1.69, respectively. Contrastingly, female ASDR and ASMR average annual percentage changes were 1.07 and 0.87, respectively. Except for a few periods of significant decrease in females, all other periods indicated a significant increase or nonsignificant changes. Incidence of IS linked to a diet high in red meat rose sharply with age, displaying increasing period and cohort effects in ASDR. Female ASMR period and cohort effect ratios initially increased and then decreased, whereas the male ratio showed an upward trend.

**Conclusion:**

This study comprehensively analyzed epidemiological characteristics that indicated a marked increase in mortality and DALYs from IS attributable to high red meat consumption, contrasting with a global downtrend. This increase was more pronounced in males than females. This research provides valuable insights for enhancing IS prevention in China.

## Background

Stroke is the critical cause of death and prolonged disability across the globe, securing the second spot in global rankings, occupying the foremost position in China, and represents the third most significant cause of combined death and disability as of 2019 (1, 2). Ischemic stroke (IS) constitutes over 80% of all stroke subtypes, representing the majority of incidence (3). The 2019 Global Burden of Disease Study (GBD) indicates that China witnessed approximately 2.87 million new cases of IS, resulting in 1.03 million deaths (47% of all stroke-related deaths), and this trend is rising sharply (4). Estimating temporal trends in the IS burden is critical for guiding disease interventions and improving public health. Additionally, previous studies by GBD collaborators have shown that approximately 90% of stroke disease burden is attributable to important environmental and lifestyle risk factors (5). Previous studies have confirmed that a diet high in red meat is a critical risk factor for IS, leading to a consistent increase in deaths and disability-adjusted life years (DALYs) globally (6). China has the world’s highest number of IS deaths associated with high red meat intake (2, 7, 8). Nevertheless, the epidemiological features and evolving patterns of IS associated with high red meat consumption in China are not well understood.

The GBD offers a distinctive framework for evaluating disease burdens and assorted risk factors, harnessing the capabilities of disease surveillance, health administrative records, vital registration systems, and additional data sources (9, 10). The objective of the study was to furnish strategic insights for China’s healthcare policymakers, thereby enabling advancements in the prevention of IS through the enactment of robust public health strategies.

## Methods

### Study data

GBD 2019 sought to deliver a uniform comparative evaluation of stroke burden and its associated risk factors spanning three decades, from 1990 to 2019, with a focus on chronological and geographical patterns as well as stratifications by sex and age. As a comprehensive tool, it facilitates the quantification of the burden attributable to hundreds of diseases, injuries, and risk factors. The data underpinning this analysis can be accessed on the Global Health Data Exchange website.^1^ In this study, we assessed deaths and DALYs from GBD 2019, along with age-standardized mortality rates (ASMR) and age-standardized DALYs rates (ASDR), to assess the disease burden of IS linked to a diet high in red meat in China. Furthermore, we tracked temporal trends associated with this burden. DALYs encapsulating a composite metric of nonfatal and fatal health losses allowed for a robust understanding of the comprehensive impact of this dietary risk. The GBD methodology for data sourcing and measurement has been delineated extensively in previous studies (9). We stratified all indicators by age, dividing patients into 15 groups (one age group per 5 years, from 25 years to 95 plus years) and sex. Ethical approval was not required because the GBD database contains public data, and no personal information was collected.

### Definitions

According to criteria established by the World Health Organization, a stroke is characterized by the rapid onset of clinical signs indicative of cerebral dysfunction that persist for longer than 24 h or culminate in death (11). In the scope of GBD 2019, IS is characterized by any vascular event causing a restriction of blood supply to brain tissues, leading to infarction. This encompasses thromboembolic and atherosclerotic strokes and notably excludes intracerebral hemorrhage. IS cases were identified using the International Classification of Diseases, Tenth Revision (ICD-10) codes G45–G46.8, I63–I63.9, I65–I66.9, and I67.2–I67.848, along with I69.3–I69.4 (12). The age-standardized rate is a statistical measure calculated to reflect the rate of occurrence normalized to the age distribution of a standard population. This adjustment facilitates the comparison of mortality and DALYs rates with greater precision by neutralizing the impact of varying demographic structures, thereby allowing for consistent comparisons over time and among different age cohorts. Age-standardized rates calculated based on the global population structure allow accurate comparisons within a country or territory. To determine high consumption levels, the mean daily intake of red meat, encompassing beef, pork, lamb, and goats but excluding poultry, fish, eggs, or any processed meat, is considered excessive if it exceeds a specified threshold. In GBD 2019, the theoretical minimum risk exposure level assigned for red meat consumption was determined to be zero grams per day.

### Statistical analysis

Deaths, DALYs, ASMR, and ASDR were used as the primary metrics to assess the burden of disease in IS attributable to a diet high in red meat from 1990 to 2019. The estimated annual percentage change (EAPC) was used to assess IS ASMR and ASDR trends attributable to a diet high in red meat over the period (13). The joinpoint regression model dissected the long-term trends of ASMR and ASDR into distinct segments to identify statistically significant trends. This model was applied to estimate the average annual percentage change (AAPC) and segmented trends within the specified period, along with the magnitude of change for each segment (14).

An age-period-cohort analysis was used to assess the impact of a diet high in red meat on the age, period, and cohort effects of the IS ASMR and ASDR. This analysis decomposed the three trends, enabling decomposition calculations and providing relatively efficient estimates (15). The effect of age captures the myriad biological and societal changes that occur as individuals age. The period effect describes variations in IS ASMR and ASDR over various intervals, reflecting the impact of a high red meat diet across all age brackets. Meanwhile, the cohort effect represents changes arising from unique risk factors and environmental exposures encountered by particular birth cohorts over time. This study meticulously estimated local drifts, longitudinal age curves, and period and cohort ratio rates (RRs). Local drift pertains to the AAPC for each age group after adjustments for period and cohort variables. The longitudinal age curve depicts the age-specific rates adjusted for shifts in both cohorts and periods. Lastly, period or cohort relative risks indicate the RR for a specific period or cohort compared with a reference period or cohort once adjusted for age and the nonlinear effects of the cohort or period. The time frames of 2000–2004 and the 1960 birth cohorts were identified as the reference groups. Statistical analyses and graphing were conducted using the R programming language. Each statistical test was two-tailed, with a *p*-value threshold of less than 0.05 indicating significance.

## Results

The national IS burden attributed to a diet high in red meat in China between 1990 and 2019 is shown in Table 1. In 2019, IS deaths related to a high red meat diet reached 73,275 individuals (95% uncertainty intervals [UI]: 36,605–103,285), with DALYs of 1,976,144 (95% UI: 1,030,749–2,665,029). Furthermore, the EAPCs for both ASMR and ASDR were 1.65 and 1.58, respectively. From 1990 to 2019 among men, DALYs because of IS related to a high red meat diet increased 3.7-fold, while the number of deaths increased nearly 4.2-fold. Furthermore, in men for the period 1990 to 2019, EAPCs for ASMR and ASDR were both above 2, at 2.03 and 2.08, respectively. From 1990 to 2019 among women, the DALYs and deaths increased 3.2 and 3.4 times, respectively, with EAPCs for ASMR and ASDR of 1.01 and 1.22, respectively. The upward trend in the number of IS DALYs, deaths, and age-standardized rates attributable to a diet high in red meat was evident, with males demonstrating a faster rate of increase than females.

**Table 1:** Analysis of IS deaths, DALYs, and ASRs associated with high red meat intake and temporal trends in China, 1990–2019.

	1990		2019		EAPC
	Number (95% UI)	ASR, per 100,000 (95% UI)	Number (95% UI)	ASR, per 100,000 (95% UI)	
Both					
DALYs	572,124 (193,675, 836,205)	66.84 (21.52, 98.01)	1,976,144 (1,030,749, 2,665,092)	100.04 (53.22, 135.24)	1.65 (1.55, 1.75)
Deaths	19,070 (5,998, 28,287)	2.8 (0.87, 4.2)	73,275 (36,605, 103,285)	4.1 (1.99, 5.81)	1.58 (1.43, 1.72)
Male					
DALYs	304,790 (101,234, 462,974)	73.83 (23.23, 112.39)	1,129,287 (567,228, 1,576,870)	119.71 (60.7, 166.6)	2.03 (1.91, 2.14)
Deaths	10,395 (3,159, 16,190)	3.36 (1.03, 5.23)	43,455 (20,841, 62,876)	5.47 (2.6, 7.99)	2.08 (1.94, 2.22)
Female					
DALYs	267,335 (86,029, 400,383)	61.02 (19.53, 91.76)	846,858 (448,037, 1,185,628)	82.98 (43.68, 115.92)	1.22 (1.11, 1.33)
Deaths	8,675 (2,641, 13,315)	2.4 (0.72, 3.7)	29,820 (14,151, 43,899)	3.07 (1.42, 4.56)	1.01 (0.81, 1.22)

ASR, age-standardized rates; IS, ischemic stroke; ASR, age-standardized rate; DALYs, disability-adjusted life years; UI, uncertainty interval; EAPC, estimated annual percentage change.

**Figure 1:**
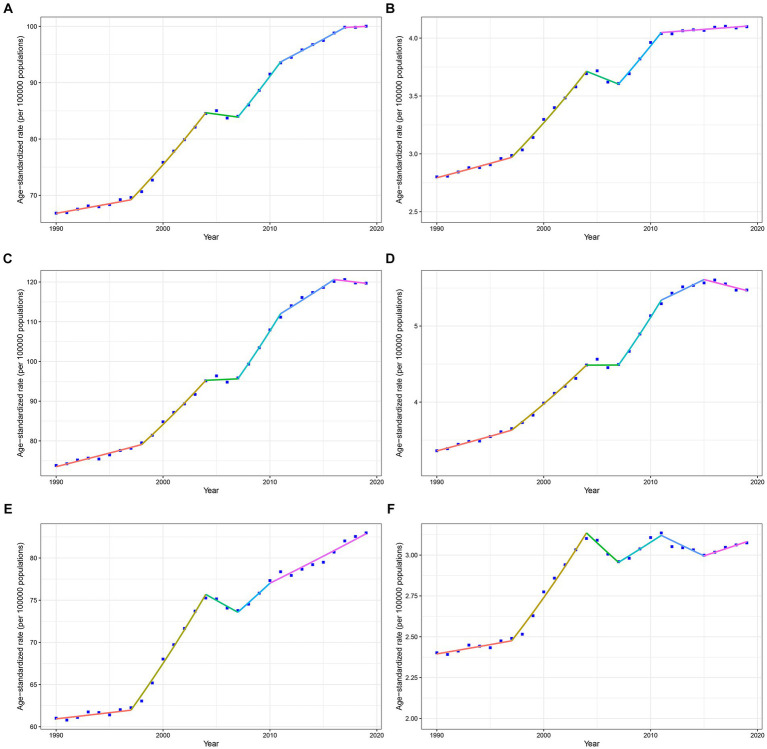
Temporal trends of IS ASDR and ASMR attributed to a diet high in red meat in China by joinpoint regression, 1990–2019. Data was provided for overall **(A)**, male **(C)**, and female **(E)** ASDRs and overall **(B)**, male **(D)**, and female **(F)** ASMRs. IS, ischemic stroke; ASMR, age-standardized mortality rates; ASDR, age-standardized DALYs rates; DALYs, disability-adjusted life years.

Figure 1 and Supplementary material illustrate the results of the joinpoint regression analysis, detailing the AAPCs in IS ASMR and ASDR associated with high red meat diets in China from 1990 to 2019. Throughout this period, both ASDR and ASMR exhibited increasing trends, with AAPCs of 1.4 (95% confidence interval [CI]: 1.25–1.55) for ASDR and 1.33 (95% CI: 1.14–1.53) for ASMR. ASDR was segmented into six epochs, whereas ASMR was divided into five epochs. Despite a few intervals showing nonsignificant AAPCs, the overall trend in most periods was upward. In men, upward trends were also identified in both ASDR and ASMR, with corresponding AAPCs of 1.69 (95% CI: 1.52–1.78) and 1.69 (95% CI: 1.49–1.9). Over the study period, male ASDR and ASMR were subdivided into six intervals, with consistent increases noted, except in the periods 2004–2007 and 2016–2019 for ASDR and the 2004–2007 interval for ASMR. Women showed AAPCs of 1.07 (95% CI: 0.82–1.31) for ASDR and 0.87 (95% CI: 0.6–1.15) for ASMR, indicating an overall rising trend as well. However, the periods of significant increases in the ASDR for women were 1997–2004 and 2010–2019, whereas marked decreases were observed in 2004–2007 and 2011–2015. In the total Chinese population from 1990 to 2019, there was an ascending trend in IS attributable to a diet high in red meat for both ASDR and ASMR, with both men and women experiencing an increase. However, the increase was more substantial in men than in women.

**Figure 2:**
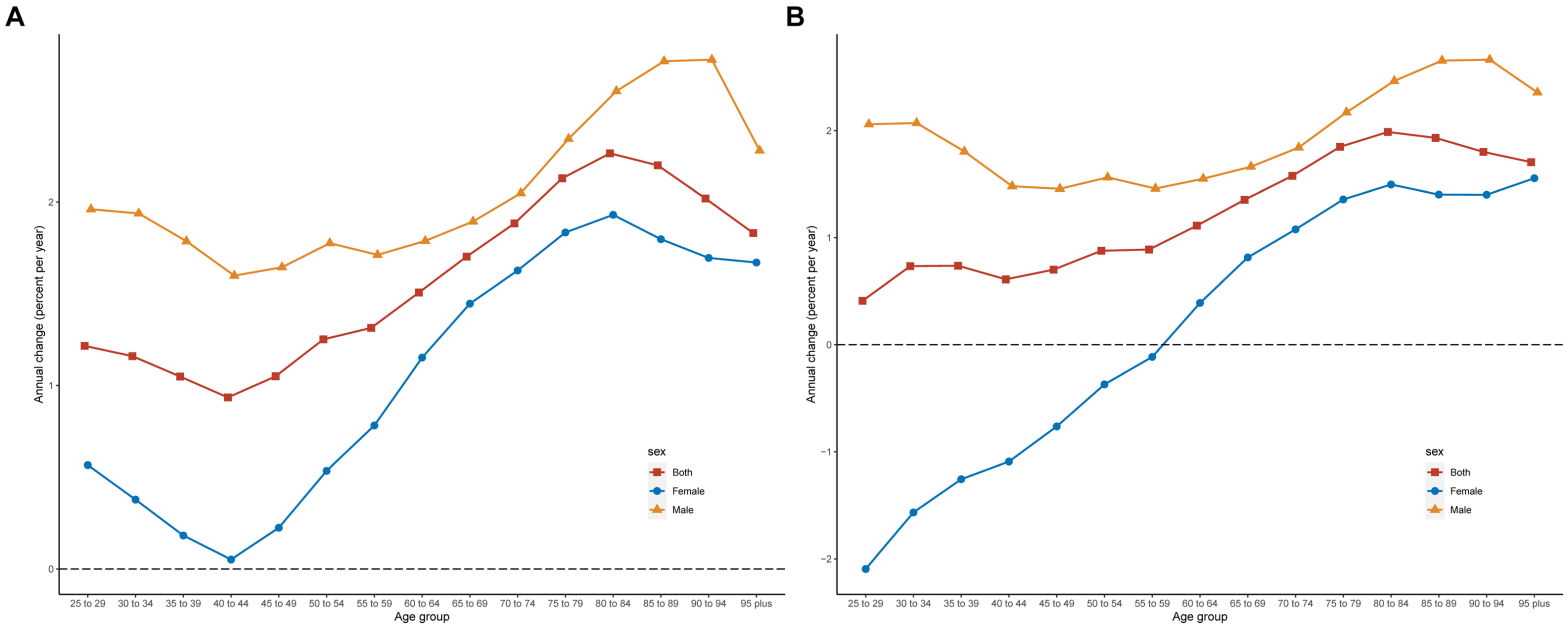
Local drifts of ischemic stroke ASDR **(A)** and ASMR **(B)** attributed to a diet high in red meat in China. ASMR, age-standardized mortality rates; ASDR, age-standardized DALYs rates; DALYs, disability-adjusted life years.

As demonstrated in Figure 2, our study further utilized age-period-cohort analysis to estimate local drifts, which provided insights into the AAPCs for IS DALYs and mortality rates attributable to diets high in red meat in China. Across all age groups and both sexes, there was a general increasing trend in DALYs rates. The local drift curves initially declined and then increased with advancing age, reaching a peak before declining. The smallest increase was observed in the 40–44 years age group, representing a minimal upward shift across all ages, whereas both the overall population and different sexes exhibited the highest increases in the 80–84 year age bracket. For mortality rates, the local drift values for all age groups were consistently above zero and showed an upward trend, particularly among males. The male local drift curve broadly followed a U-shaped distribution, with the smallest increase occurring in the 55–59 years age group. By contrast, women experienced a declining trend before the 60–64 years age range, followed by an increase, with the most significant decrease observed in the 25–29 years age group. Furthermore, regarding DALYs or mortality rates, the rate of increase in any age group was consistently lower for females than for males.

**Figure 3:**
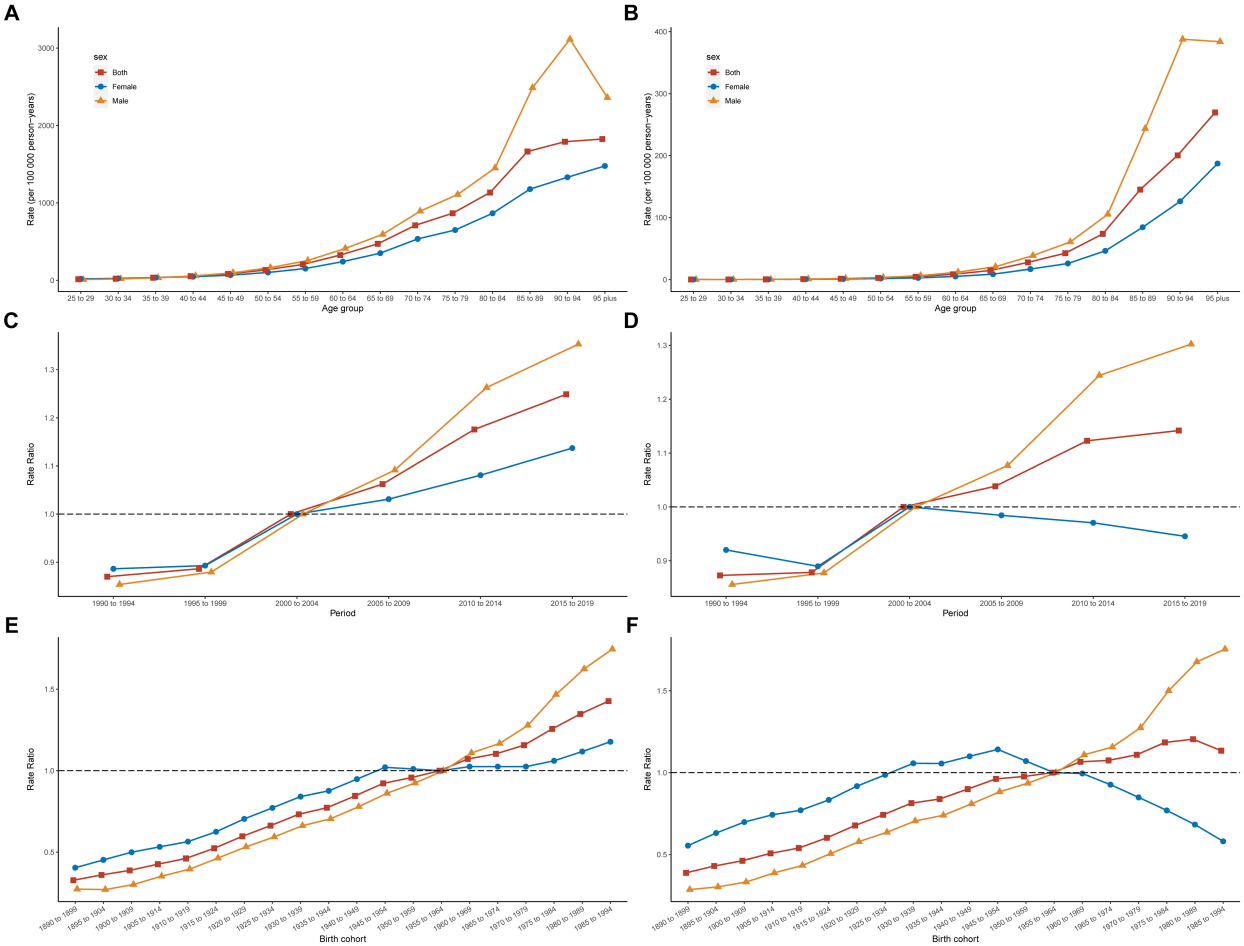
Age-period-cohort analysis of ischemic stroke ASDR and ASMR caused by a diet high in red meat in China. Data provided for age **(A)**, period **(C)**, and cohort **(E)** effects of ASDRs and age **(B)**, period **(D)**, and cohort **(F)** effects of ASMRs. ASMR, age-standardized mortality rate; ASDR, age-standardized DALYs rate; DALYs, disability-adjusted life years.

Figure 3 illustrates the estimated longitudinal age curves, period effects, and cohort effects on IS mortality and DALYs associated with high red meat consumption in China. Our research revealed that both DALYs and mortality rates significantly increased with age, and this acceleration was more pronounced among older populations, particularly within the mortality rate curves. Notably, from age 50 years onwards, the increase in both DALYs and mortality rates in males exceeded the rates in females, with the disparity becoming progressively more substantial after 80 years of age. Period effect analysis indicated a general upward trend in the DALYs rates for IS related to high red meat intake from 1990 to 2019. Before the 2000–2004 period, the RRs for males were lower than those for females. However, in the following years, there was a marked and significantly sharper increase in the male RR values than in those for females. Compared with the 2000–2004 period, earlier periods were characterized by lower mortality rates, whereas subsequent periods showed a continuous increase in both overall and male-specific RR values. For females, the RR peaked in the 2000–2004 period and was lower in all other years. Cohort effect analysis suggested an increasing trend in IS DALYs rates attributable to high red meat intake. Before the 1955–1964 cohort, female rates were higher than male rates. After this period, there was a greater increase in the RR values for males, eventually surpassing the rates in the female cohort. The overall trend in mortality rates increased for both sexes; however, for females, there was an initial increase followed by a gradual decline from the 1955–1964 cohort onwards. Pre-1955–1964, the burden for females was higher than that for males, but this trend reversed thereafter. In general, as age advances, the burden of IS attributable to diets high in red meat increases, with males experiencing a higher burden than females.

**Figure 4:**
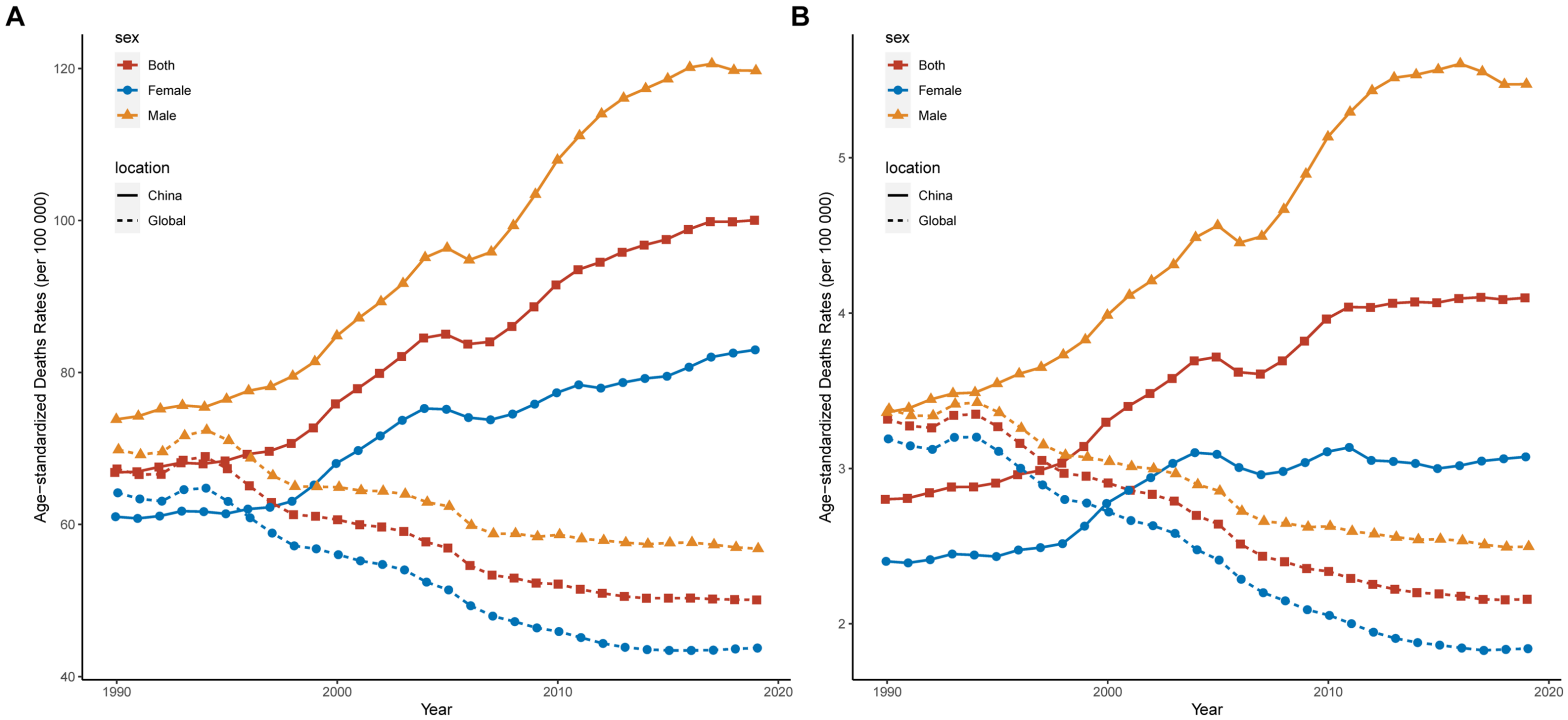
Trends in ischemic stroke ASDR **(A)** and ASMR **(B)** associated with high red meat consumption in China and globally from 1990 to 2019. ASMR: age-standardized mortality rates; ASDR, age-standardized DALYs rates; DALYs, disability-adjusted life years.

Figure 4 depicts the trends in ASDR and ASMR for IS attributed to a diet high in red meat in China compared with the global figures from 1990 to 2019. During this period, China experienced a general climb in the IS ASDR overall and for both sexes related to high red meat consumption, with a sharper increase for males than for females, surpassing the global average. Although Chinese women had a lower ASDR than the global average prior to 1996, the global female ASDR declined, while the Chinese female ASDR escalated. Overall, before 1997, China’s ASMR was below the global level, after which it began to ascend, exceeding the global average. Before 2000, females had an ASMR below the global average; however, thereafter, it exhibited a fluctuating rising trend in contrast to the declining trend at the global level. Throughout the entire timeframe, male ASMR consistently surpassed the global level, with a notably larger margin of increase. Over the past 30 years, China has seen an increasing trend in IS disease burden attributable to diets high in red meat, which is higher than the global norm, and the global picture is a downward trend. The burden among Chinese males exceeds that of females, and the decline in the global male burden is less pronounced than that for females.

## Discussion

This study offers an exhaustive overview of the most recent data on the temporal patterns of IS-related deaths and DALYs associated with a diet high in red meat, broken down by sex in China over the period from 1990 to 2019. Our findings indicate a varying degree of increase in IS deaths and DALYs associated with diets high in red meat during this period, observed both in the general population and among different sexes. In addition, RRs of various periods and cohorts exhibited clear upward trends. It is noteworthy that IS DALYs rates and mortality associated with red meat consumption increased sharply with age. Furthermore, the rise in DALYs and mortality rates among males not only surpasses that of females but also does so at an accelerating pace. The rates of IS mortality and DALYs in China attributable to high red meat intake exceed global averages, indicating the need for targeted, sustained efforts to reduce the mortality and DALY burden from IS related to such diets.

Studies have demonstrated a connection between the consumption of fresh and processed red meat, total red meat intake, and an increased risk of IS (16–19). As well, consumption of red and processed meats has been moderately linked to elevated mortality rates from all causes, as well as from cancer and cardiovascular diseases (20–22). A diet high in red meat may contribute to the development of hypertension, obesity, vascular sclerosis, and hyperlipidemia, factors that potentially elevate the likelihood of fatal IS (23). Numerous risk factors linked to IS, including oxidative stress, the generation of free radicals, lipid peroxidation, vascular inflammation, and atherosclerotic processes, play crucial roles in its development (24). For instance, the saturated fatty acids found in red meat correlate with elevated levels of low-density lipoprotein cholesterol in the bloodstream (25). In addition, the heme iron content in red meat may be associated with oxidative stress (26). Research within a UK cohort established a distinct metabolic and mechanistic connection between the consumption of unprocessed red meat and IS mortality. This suggests that triglycerides derived from lipoproteins, fatty acids, and various non-lipid metabolites may be pivotal in this relationship (27). One study suggested that consuming white meat may reduce the relative risk of stroke (20). Substituting fish for red meat has been found to reduce the risk of strokes resulting from atherosclerosis in large arteries as well as the risk of occlusion in small blood vessels (28).

Age is the most potent and unmodifiable risk factor for IS, with a higher incidence and worse functional recovery in older individuals, leading to higher mortality and DALYs rates in this demographic population. The increased age-standardized deaths and DALYs rates for IS in the overall population of China can largely be explained by demographic shifts, such as aging. In 2019, China’s population aged 65 years and above was 165 million, with individuals aged 80 years and above totaling 26 million (29). Although China’s birth policies have changed, the aging trend is unlikely to be reversed in the short term.

Studies affirm that the EAPC for IS attributed to high red meat intake substantially decreased globally from 1990 to 2019, but it is the second-highest behavioral risk factor after smoking in younger populations (30). Between 2013 and 2018, red meat consumption increased among Chinese adults, with the proportion consuming 100 g or more daily increasing by 10%, and male and female proportions of 49.7 and 34.9%, respectively (31). One study found that the IS disease burden attributed to a diet high in red meat in China is increasing, which may be related to changes in dietary habits among the Chinese population (7). As China undergoes economic development, dietary and lifestyle changes have increased the population’s exposure to risk factors for IS. Our analysis of the GBD data have demonstrated a higher burden of IS associated with high red meat diets in men than in women, with men exhibiting noticeably higher ASMR and ASDR. Beyond the fact that men consume more red meat than women, sex differences in risk factors such as obesity, smoking, high blood pressure, and differences in lipid metabolism might contribute to higher mortality rates in men. It is important to note, however, that related studies have found that females are less likely to receive a diagnosis and treatment for IS compared with males, and that females receive less social support than males (32, 33). There are also stroke risk factors specific to females including oral contraceptive use, other hormone use, pregnancy, and menopause (34, 35). Therefore, further research is needed to develop gender-specific dietary guidelines.

The primary risk factors for IS are behavioral (36). One study showed that subsidies for healthy foods, such as fruits, vegetables, whole grains, and nuts/seeds, and taxes on processed meat, unprocessed red meat, and sugary drinks reduced cardiovascular disease mortality (37). Physical activity remains a powerful means of lowering the IS mortality risk from meat intake; however, the overall consumption of red and processed meats, particularly the latter, should be controlled (38). Primary prevention of IS includes lifestyle and dietary changes and treatment of risk factors such as high blood pressure, diabetes, and lipid disorders (39–41). A prospective cohort study of half a million middle-aged and older Chinese individuals demonstrated a significant reduction in IS risk with a healthy lifestyle including lower red meat intake, active exercise, and higher vegetable consumption (42). However, a higher intake of red meat has been independently predicted to lead to cardiovascular disease among urban and high-income inhabitants, although not among the poor, with red meat intake having a protective effect against cardiovascular diseases and all-cause mortality in rural and low-income residents (43). Thus, China should allocate more targeted resources to prevent and treat IS in older individuals with lower economic status (44).

This study indicates that, compared to other countries, there is significant room for improvement in China in the prevention and control of IS attributable to diets high in red meat. Hence, it is imperative to raise public awareness in China about the harmful effects of a high red meat diet on the risk of IS and to establish scientific dietary plans. Transforming dietary habits might seem formidable, yet the government has the capacity to facilitate this modification across the populace via a multifaceted strategy. This strategy could encompass educational initiatives and awareness drives, regulatory measures and legislative reforms, fiscal encouragement and subsidies, comprehensive public health endeavors, and collaborative efforts with private entities (45).

While our study has yielded meaningful conclusions, its limitations should also be considered. First, the dietary red meat intake of Chinese residents was primarily estimated through diet questionnaires, which could introduce biases affecting the validity of the fiber intake estimates. Second, although death registration data were obtained from civil registrations and large cohorts, their coverage and representativeness may be flawed. Third, our study is based on the entire Chinese population, and many meaningful variables, such as sample origin and economic status, cannot be assessed, thus preventing a more in-depth analysis. Fourth, our analysis did not differentiate between the impacts of various red meat categories on the IS disease burden. Therefore, it is necessary to implement large-scale, multicenter randomized controlled studies in the future to clarify the effects of a high red meat diet on IS in China.

## Conclusion

In conclusion, this study identified a significant increase in IS mortality and DALYs associated with a high red meat diet in China from 1990 to 2019. The increase in these figures was notably higher among men than women, and the upward trend in China was more pronounced than that in the global context. These findings underscore the importance of implementing measures in China to reduce the intake of high red meat diets and highlight the necessity for more effective strategies aimed at the prevention and treatment of IS, especially focusing on the male population.

## Data availability statement

The original contributions presented in the study are included in the article/Supplementary material, further inquiries can be directed to the corresponding authors.

## Author contributions

SJ: Data curation, Formal analysis, Methodology, Software, Writing – original draft. KX: Conceptualization, Data curation, Methodology, Writing – original draft. BS: Formal analysis, Methodology, Visualization, Writing – original draft. LX: Investigation, Project administration, Supervision, Validation, Writing – review & editing. HZ: Conceptualization, Funding acquisition, Project administration, Supervision, Validation, Writing – review & editing.

## Footnotes

^1^ http://ghdx.healthdata.org/gbd-results-tool
